# Impacts on quality: Enjoyment factors in blind and low vision audience entertainment ratings: A qualitative study

**DOI:** 10.1371/journal.pone.0208165

**Published:** 2018-12-03

**Authors:** Mala D. Naraine, Deborah I. Fels, Margot Whitfield

**Affiliations:** 1 The Centre for Research on Work Disability Policy, Toronto, Ontario, Canada; 2 Inclusive Media and Design Centre, Ted Rogers School of Information Technology Management, Ryerson University, Toronto, Ontario, Canada; Tsinghua University, CHINA

## Abstract

Audio description (AD) is one of the main methods that people who are blind or low vision (B/LV) use to access film, television, and theatre content. AD is a second audio track inserted into the space(s) where speech is absent, which tends to be only a few seconds. Contained in that second track is an audio description of the important visual information contained within a specific scene. However, as there is insufficient time to describe all visual information, decisions about what is important to describe and how to present that information (style) to optimize a B/LV viewer’s entertainment experience are required. Most research to date has considered only short-term, single-episode experiences to gauge viewers’ reactions to the AD content. In addition, this research typically has used a monotone, single style of audio description, which is defined as “the conventional style” in this paper. We use an integrative style instead, that is defined as ‘AD designed to fit a specific show”, and differed between shows. We carried out a within-subjects longitudinal study with eight episodes of a dark comedy, using different description styles and describers in order to assess viewer engagement and preferences for AD describer style, language use, timing, and fit to the show. Twenty-four blind participants viewed and rated all eight episodes. Major findings included that most participants found the integrative style entertaining, a fit with the specific episodes, and enjoyable. Some participants, however, preferred the conventional style and struggled with the language and topic of a dark comedy and its associated descriptions.

## Introduction

Blind and low vision (B/LV) children and adults of all nationalities continue to experience barriers to accessing many of their own cultural artefacts, due to the lack of access to audio description (AD), a secondary sound track produced to provide description of important visual elements of media content for people who cannot see [1, 2]. Cultural access is restricted when media is inaccessible, further excluding B/LV people in society [3], despite ongoing advocacy for AD to be considered a human right and a normal expectation for humans [4, 5]. Without access to AD and other forms of assistive media technologies, “the gap widens between a person’s abilities and the sociocultural perception of their disabilities” [6]. Discriminatory perceptions of B/LV people have resulted in exclusion of B/LV people’s experiences and contributions to past AD research. However, most current AD research is taking account of B/LV audience needs and preferences [7, 8, 9, 10, 11], but there is fairly limited research into B/LV feedback on methods of AD production in televised media content, and even fewer longitudinal AD studies have taken place with B/LV audience feedback [12].

Canadian B/LV people have only recently had access to television and film with formal AD. Canadian guidelines and regulations were established in 2004 by the Canadian Radio Television and Telecommunications Commission (CRTC) to facilitate the development of accessible entertainment media [13]. B/LV individuals, however, did not contribute to the development of those AD standards. Meanwhile, Canadian media, including televised and nationally broadcast Canadian cultural content, has been evolving [14] in a decentralized form, like all media. B/LV viewers are continuing to experience barriers [3, 15] to accessing cultural media content, thereby potentially limiting their digital competency [16]. There has been little empirical research in this area of media and cultural literacy for B/LV adults with respect to entertainment, despite its substantial impact [6]. This impact extends not only to digital technological competence but also, as outlined in Alper’s [17] paper, important lifelong metacognitive skills for critical thinking, such as play, performance, appropriation, judgment, negotiation, and visualization. Further research is needed in understanding media competency in B/LV adults, as well as how B/LV adults perceive subversive and emotional content designed to disrupt social norms [18, 19, 20].

In this paper, we will present a longitudinal assessment of an eight-part television comedy, *Death Comes to Town*, produced by Bruce McCulloch, directed by Kelly Makin, and distributed by the Canadian Broadcasting Corporation. The description was created using a Canadian integrated model approach [2] that combined AD delivered with appropriate emotion and intonation, fitting to the content being described, as opposed to a “newsreader” style delivery, inconsistent with the breadth and diversity of media content today. The research questions are: 1) what is the longitudinal impact on B/LV audiences of the Canadian integrated model of description for an eight-part television comedy; and 2) what are the positive and negative factors identified by users for the AD and the show? We hypothesize that the longitudinal enjoyment of the show’s and the quality of the AD (as assessed by viewers) will be positive and that there will be a positive response to the integrated AD related to fit with the show style and describer’s voice characteristics such as pace, language and emotional match. We examine and report on the impact this Canadian-originated approach [2] has on Canadian B/LV viewers over time, considering the emotional impact on B/LV viewers and their responses to this AD approach.

## Background

### AD and comparable access to entertainment media

Blind and low vision access to media content, comparable to that which sighted audiences have, means unprejudiced and direct content access through an AD delivery style that suits the content which it is describing (similar to what a Deaf person would expect of his or her oral-to-signed translator) [2, 7, 10, 19]. AD is part of how a show is delivered to and consumed by B/LV audience. While we are using this definition to underpin this paper, others define it differently. For example, some authors refer to comparable access to entertainment as an “equivalent engaging experience” [8, 19, 21]. Others, may consider comparable access from a technical point of view such as providing only the raw data or facts of the visuals and the B/LV must interpret that ….” [10]. A sighted audience does not receive the facts of show but a director’s interpretation of it in all manners (e.g., costumes, set, lighting, actor intonation, etc.). As such, comparable access is about translating the director’s vision and style for the visuals of a show into an auditory equivalent, or additional layer, given the limitations of restricted time and opportunity to add AD. Naraine et al., [12] examines a case study where the director/describer’s artistic approach to AD was successful in achieving the integrative style, which allowed B/LV to have a comparable entertaining experience to sighted theatre goers. Lopez et al., [21], further contends that issues of accessibility should be incorporated into film and television to facilitate an audio track that is represents the creative vision which could then also be made available to all audiences. Thus, they argue that “the diversity of preferences by visually impaired people cannot be reduced to one accessibility method, but on the contrary requires a user-centred personalised method that allows audiences to make choices on access strategies” (p. 4). The fundamental objective of AD, through the delivery style or method of the access strategy, is to allow B/LV audiences to have a comparable entertaining experience.

Standard style AD, as defined by some existing standards such as OfCom [22], has restricted the role of the describer to that of a neutral newsreader, delivering all content, even horrific or hilarious content, in a neutral and emotionless manner. Historically, an AD describer was restricted to adhering to a fairly strict set of language and phrasing guidelines [22].

The Canadian-derived integrated model or integrated approach to AD [2] has been influential in challenging the mindset of the ingrained newsreader-style approach to AD, which discouraged creativity in AD content development and emotional expression in AD content delivery. The rationale for this unique Canadian approach stemmed from the idea that B/LV viewers should have a similar experience to media content as their sighted peers; if all AD content always followed a similar approach in its development and delivery, then B/LV audiences would have, not an comparable, but rather a consistent experience of their media [7, 23]. If all B/LV viewers experience their media access in a consistent manner, they will be sheltered from media content differences (thereby limiting their media literacy competency) as well as denied access to the entertainment value of that content.

Since this early work, other researchers throughout the world have further explored alternative approaches to conventional AD for a variety of genres. For example, Walczak et al., [7] explored presence and engagement for a naturalistic drama in English using creative description that is driven by the terminology and style of the film. Szarkowska et al., [24] uses a description style for Polish content called “auteur description” that follows the director’s vision, rather than some unrelated style. This research has collectively found that B/LV not only prefer the alternative styles, but are also more present and engaged in the content, compared with the standard AD style. However, most research relies on a single show or episode to draw conclusions. In this paper, we present data from an entire 8-episode comedy, where the AD is presented with different styles and describers over the series, to explore whether the potential novelty effect of a single viewing will degrade B/LV assessment and engagement in an alternative style of AD.

### Entertainment theory

Television and film are media whose main purpose is entertainment [24]. The director is responsible for meeting this goal by providing the vision and general structure of the content. All other components of the structure, such as script/screen writers, actors, costumes, sets, editors, etc., are drawn together to meet that vision.

What makes a film or video entertaining is not simply the visuals or dialogue, but rather the aggregate of complementary sensory elements [25]. For instance, the aural narrative can enhance the visual narrative [26], and vice versa. Non-diegetic sounds, such as the sound of Grinda’s Caribbean ring tone or the stereotypical horror music accompanying the death of the mayor in *Death Comes to Town*, are used for dramatic effect, humour, and emotional appeal.

### Director’s role

The director decides every aspect of a film, such as music, lighting, costuming, and camera lenses, from pre-production to post-editing [27], all the while taking into consideration the audience’s expectations and overall entertainment experience [2]. Although the director holds the ultimate decision making power, filmmaking is still a collaborative and creative process [28], consolidated through the director’s vision, leadership and observations.

Dialogue in film is rich in meaning and can range from a form of communication to an “aesthetic effect” [29]. Viewers (including B/LV people) immersed in the story develop specific expectations that stimulate their emotional responses and facilitate the formation of meaning [27]. Directors strive to stimulate specific emotional responses from their viewers, as an emotional connection to the film and the characters within it help drive the entertainment experience of the audience [30]. For example, someone watching *Death Comes to Town*, whether B/LV or sighted, wants to be engaged and entertained: Within this context, entertainment could be defined by how amused or frightened the audience is during the episode or scene. A person who is B/LV experiences the emotion and forms meaning through all of the audio elements, including the AD.

Traditional AD practices have not enabled director involvement in the process of AD creation, as it usually occurs once the entire production has been completed, and often by a third party who has no involvement in the show’s production. As a result, the director’s vision can easily be misinterpreted, potentially limiting or confusing the entertainment experience of B/LV audiences.

Romero-Fresco [31] suggests that filmmaking should also include audio-visual translators such as audio describers and captioners, in order for the process to be more accessible. Hence, it would be necessary for the director to oversee the AD development, controlling (as much as possible) the entertainment outcome and responses of B/LV viewers. Without the director’s control over the AD process, she cannot convey her interpretation of the film’s content [2]. Audio description should thus be incorporated in the creative process rather than potentially compromising the entertainment experience of B/LV viewers. The creative team, under the leadership of the director, is in the best position to make the necessary adjustments required to ensure a rewarding and enjoyable experience for B/LV viewers [2, 31]. Some directors have already begun to implement AD in new and creative ways [32], exploring open, subjective, as well as artistic forms of AD [11]. The director’s creative influence over the implementation of AD strategies fits in with principles of universal design because it is integrated at the beginning of the process and the designer of the product is involved in the process [2].

### Comedic style and death Comes to Town

Comedy is a genre of film that lends itself to entertainment value. In French philosopher Henri Bergson’s essay, *Laughter*: *An Essay on the Meaning of the Comic*, Bergson attempts to grasp the basic building blocks of comedy [33]. For Bergson, comedy is derived from our shared social and cultural habits *de jure* (of the day) and from our ability to imagine animate beings as inanimate or mechanical objects. For example, the inflexibility and mechanical movements of mimicry naturally make us laugh because we imagine the body as a machine and not as a sentient being.

*Death Comes to Town* is a murder mystery combined with comedy series written by comedy troupe “Kids in the Hall”. The mayor of the town of Shuckton is murdered, and everyone in the town becomes a suspect. The Grim Reaper also attempts to collect more souls as the mystery unfolds. The series presents various elements of comedy, including vulgarity, irony, parody, and perverse humour. The English wit of this comedy presents itself in paradoxical figures of speech (e.g., “things are going forward in a forwardly direction” and “I didn’t do nothing that I can’t remember”), ridicule (e.g., “your make-up needs make-up”), and sarcasm. The bizarre incidents that plague the quirky characters of the series, combined with the verbal comedy of the writers and the acting, provide a funny and entertaining experience for audiences.

Makin adopts some of Bergson’s theoretical underpinnings in the show’s dark comedy, an elusive sub-genre of film often presenting contentious or taboo themes, such as death [34, 35], not typically found in traditional comedies. For example, one of the characters is found dead outside his house standing with a 90^0^ angle bend in his body and his head stuck in a mailbox (an unconventional and mechanical position to find someone in). The tragic and comic impulses are juxtaposed against each other: The tragedy of the murder is offset by the comedic character–flawed weather reporter Heather Weather–who proceeds with flagrant insensitivity to report on the death, tying it into her report on the weather.

## Method

Blind and low vision Canadians from Toronto were recruited to participate in a study on AD where the integrated model approach [2] to AD was employed for *Death Comes to Town*. Participants volunteered and were selected on a first-come, first-served basis. They were asked to complete a pre-questionnaire before watching the shows. They were required to watch all eight episodes in the series and complete a short questionnaire after each episode, as well as a post-questionnaire once all eight episodes were viewed. Participants received the shows on DVDs or CDs by mail and either completed the questionnaires online using a survey tool or they were sent a hard copy format of the questionnaire by mail. Two participants opted to carry out the study on campus, while all other participants worked at their own pace at home over a three-month period. Blank questionnaires can be found in S2 Appendix. This paper will report on the results of the between-episode and post-study questionnaires.

Twenty-four B/LV individuals participated in this study. A pre-questionnaire was used to obtain demographic information and experience with AD. Thirteen participants self-identified as blind (no functional vision), and 11 self-identified as low vision (limited functional vision). Eleven participants indicated that they were congenitally blind, and thirteen indicated that they were adventitiously blind. The age range of participants included: four “between 19 and 29”; seven “between 30 and 39”; nine “between 40 and 49”; two “between 50 and 59”; and two were “60 and over”. Participants varied in their levels of experience with AD: 20 were “very familiar” or “familiar”; two were “somewhat familiar”; and two were “not familiar”.

The questionnaire used between episodes consisted of seven questions: Question 1 required that participants indicate their participant identification number. Questions 2 and 3 asked participants to rate their enjoyment level of the show and of watching the shows with AD (on a five-point Likert scale); Question 4 asked participants to rate on a ten-point scale the quality of the AD where quality was a subjective assessment from the participant. Question 5 asked participants to identify the positive factors that affected their rating of the AD including: “the describers pace” and “tone of voice”, “language and vocabulary”, “quality of description, such as fit of description to show”, and any additional factors. Question 6 asked participants to identify the negative factors that affected their rating of the AD and used the same categories as question 5. Question 7 asked participants to provide additional comments about the show and the AD.

The post-questionnaire consisted of 15 questions: three questions asked participants to rate on a five-point Likert scale the level of entertainment from watching the shows with AD; eight questions asked participants to rate on a five-point Likert scale the level of agreement with statements pertaining to enjoyment of the type of show and the quality of the AD, including style, speed, and quantity of information conveyed by the AD. One question asked participants to state if they were likely to purchase an audio-only track of their favorite television show. Three open-ended questions asked participants to reflect on their entertainment experience with the AD for all eight episodes of the show. The questionnaires were based on ones developed for research on AD and used in five other studies [36, 37, 38, 39, 40] providing a measure of external validity. The between episode questionnaire used only the AD quality measures from the previous questionnaires.

Data were analyzed using repeated measures statistical analysis as well as qualitative analysis. Themes (see Table 1) were developed from the open-ended questions. Twenty percent of the open-ended question data was then categorized into the various themes by two independent raters to measure reliability of the themes, using an Interclass Correlation (ICC) statistic. The ICC was above 0.6 for all themes, so one rater analyzed the remaining open-ended question data.

**Table 1:** Themes for the open-ended questions in the between-episode questionnaires.

Theme	Definition
Technical	Comments related to the sound quality of the audio, volume level, difference between original sound track and description track, synchronization between two tracks.
Audio description style	Comments related to like/dislike of style of narrator, tone of voice, difference between characters and narrator, fit of AD to style of show (e.g., humorous and so on).
Audio description–general	Comments related to like/dislike of AD in general, including level of distraction of the AD to the show itself.
Pace	Comments related to how fast the narrator was speaking (“too fast” is considered negative, “good pace” is considered positive).
More character details	Request to have more character-related information, e.g., clothing, facial expressions, personal characteristics, or identifying missed items relating to characters.
More setting details	Request for more detail related to setting, e.g., description of scene changes, scenery, set, and weather, or identifying missed items relating to setting.
More action details	Request for more details related to action, plot, or activities being performed, or identifying missed items relating to action.
Not enough details—general	Request for more detail in no specific area.
Language	Request for different language other than that used by the describer.
Show	Statements about enjoyment or dislike of the show.

### AD and Death Comes to Town

*Kids in the Hall*: *Death Comes to Town* was broadcast on CBC Television between January 12 and March 16, 2010. Members of *The Kids in the Hall* play all of the major characters within the series.

The integrated method was used to produce the *AD for Death Comes to Town*. The AD was added in post-production, with the executive producer taking an active role in the AD strategy. The film editing company, Deluxe, was contracted to produce the AD, which they outsourced to a private AD company, Exposure TnT. The research team collaborated with Exposure TnT, acting in an advisory capacity to facilitate and integrate the integrated style of AD. Exposure TnT used three actors trained in the integrated style of AD as the voice talent. Different actors described different episodes as follows:

1. LC (describer in episodes 1, 3, & 5) performed in numerous theatre productions and operas. He also acted in film and television series, including a role as the principal in *TAKEN 'High Hopes'*.
2. BY (describer in episodes 2 & 4) played the principal in a number of films and television shows. He has also acted in various theatres throughout Canada and abroad.
3. SC (describer in episodes 6, 7, & 8), has a breadth of experience in theatre, film, television, and radio. From stage combat fighting to various dialects, SC has varied interests and skills.

Three different actors were used to examine differences between the actors’ approaches. In *Death Comes to Town (described)* B/LV viewers are exposed to three different styles in terms of dramatic emphasis as a result. Dramatic emphasis is crucial in AD as it is the only vehicle the describer has to translate visual stimuli to audio stimuli. Dramatic emphasis is also essential in AD as it is salient to whether or not B/LV viewers become engaged in the show and thus, it impacts the entertainment value experience for this audience [2, 7, 8, 10, 19, 23, 36]. AD in the following three episodes were analysed for dramatic emphasis and style.

#### LC Episode 1

LC was dramatic and melodramatic. His voice portrayed elaborate emotional emphasis as he was very expressive at all times. His AD consisted of straight forward description of the action. The AD on settings is supplemented by emotive commentary such as “ridiculously large jock strap”, “wrenches the mike”, and “obese man is disappointed.” He used humour and irony, e.g. “Stroking her pussy … cat.” He expressed excitement when the airmail letter was arriving, mirror in the excitement of the crowd. LC’s melodramatic style was consistent and fit the style of the show.

#### BY Episode 2

BY was not as dramatic as LC but he expressed a bit of dramatic emphasis when the mayor was found with his head in the mailbox. Expressions of elements of dramatic emphasis emerged in his voice through sarcasm when describing the cops eating ice cream. Another example of dramatic emphasis portrayed by BY is when Death is dancing outside Ricky’s house, which gives the blind viewer the sense that something strange is happening. He was a moderately good describer in terms of dramatic emphasis.

#### SC Episode 8

SC portrayed little or no dramatic emphasis. He had a tendency to resort back to the newsreader style. He sometimes did not speak in complete sentences. His sentence fragments did not match the style of the show. When there are dramatic scenes, he raises his voice instead of changing the style. His AD improved during the scene when the lawyer and nurse are puckering to kiss in the vet’s office. Towards the end of episode 8, he gets more emotive, with comments such as “He hangs on for dear life”, “It’s a bumpy ride”, and “Big City is orgasmic!” Although his AD slightly improved, his AD style in terms of dramatic emphasis was not as consistent as LC and BY.

Blind and low vision audiences were asked to evaluate the quality of the AD for each episode and for the entire series as a whole. This study was approved by the University’s Research Ethics Board and participants provided written consent prior to beginning the study.

## Results

### Between-episodes questionnaire

A repeated measures ANOVA was used to assess the level of enjoyment of the AD between the episodes, and the quality rating of the AD. For level of enjoyment of the episodes and of the AD, Mauchley’s measure of sphericity was significant, and as a result, the Huynh-Feldt correction (epsilon = 0.51) as the test for significance was used. There was no significant difference between episodes for ratings of enjoyment [F(7,140) = 0.84, p>0.05, partial eta = 0.041] or of the AD [F(4,133) = 1.5, p>0.05, partial eta = 0.073]. For ratings of quality of the AD, Mauchley’s measure of sphericity was met. There was also no significant difference between episodes for quality of the AD [F(7,133) = 1.21, p>0.05, partial eta = 0.06].

**Fig 1:**
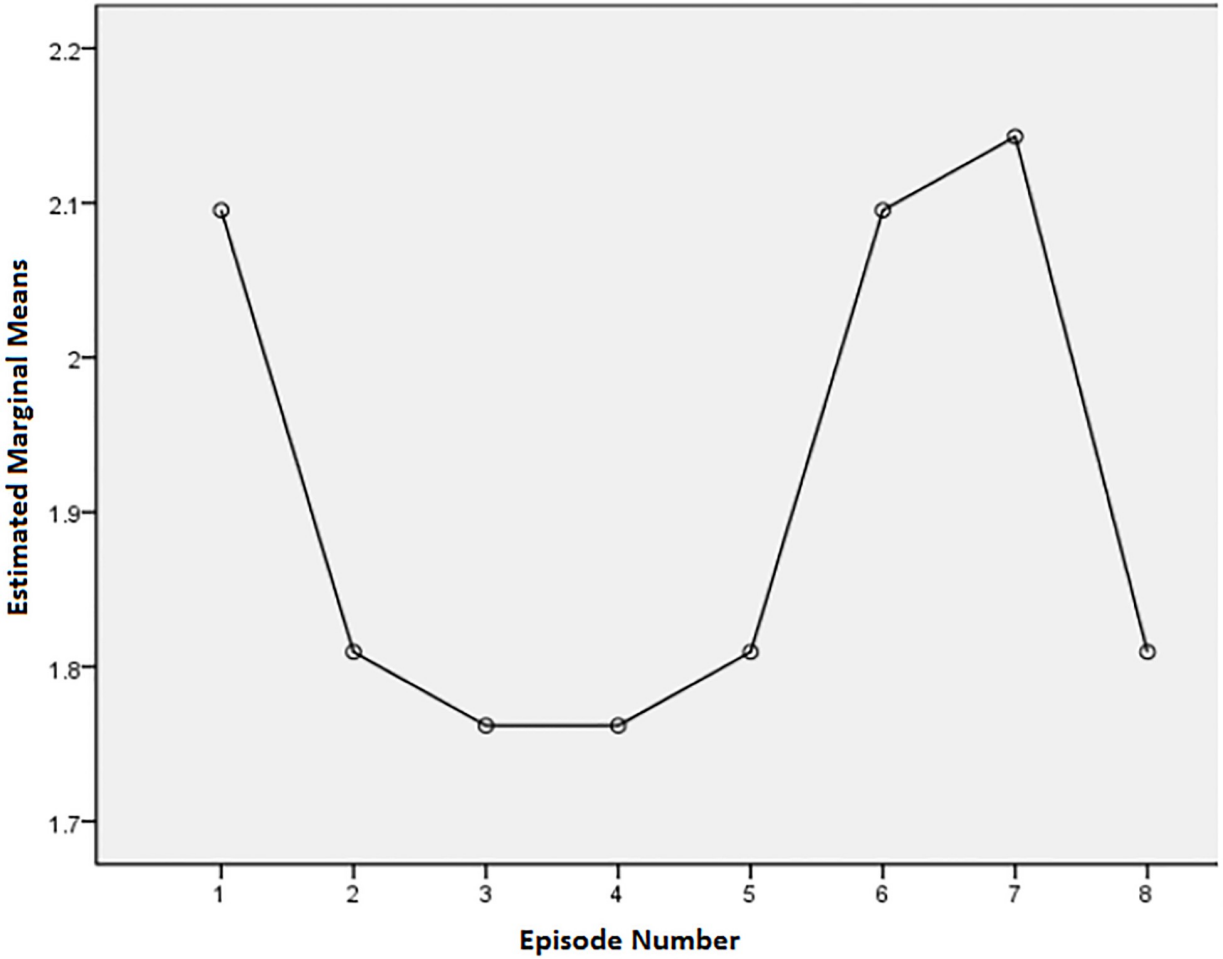
Means plot for enjoyment of show ratings (1 is very enjoyable and 5 is not enjoyable at all).

**Fig 2:**
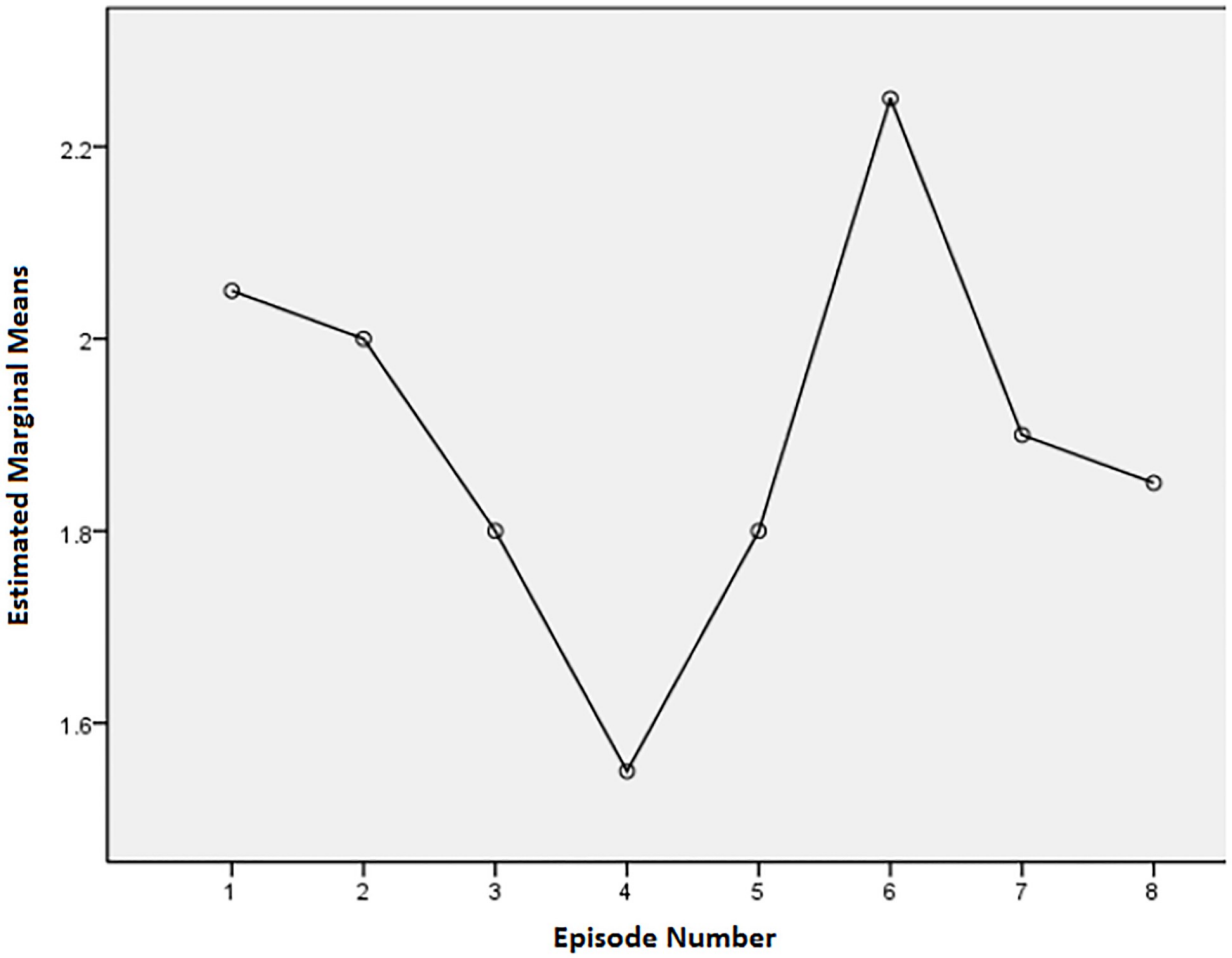
Means plot for enjoyment of the AD ratings (1 is very enjoyable and 5 is not enjoyable at all).

**Fig 3:**
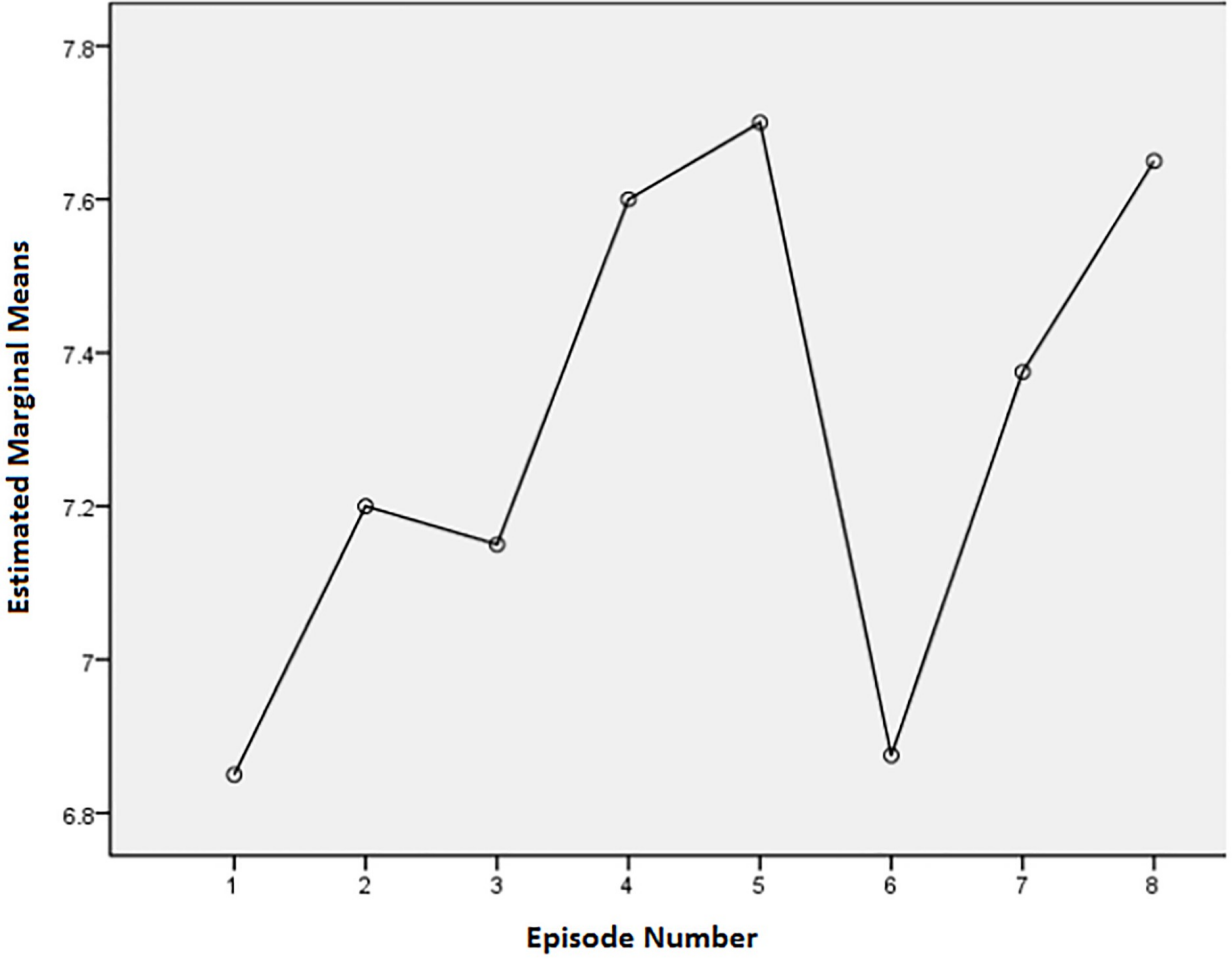
Means plot for AD quality (1 is low quality and 10 is high quality).

Although there were no statistically significant differences between episodes, there were some noteworthy descriptive trends. Examining Figs 1–3, there are some obvious patterns. Episodes 3, 4, 5, and 8 (one each from describer 1 and 3, and two from describer 2) always had the most positive scores of the eight episodes. For the question about the level of enjoyment of the show, episodes 4, 5, and 8 had means of 1.76 (SD = 1.31), 1.81 (SD = .98), and 1.81 (SD = 1.12) respectively, where a score of 1 is the most positive response. The level of enjoyment of the AD means for episodes 4, 5, and 8 were 1.55 (SD = 0.83), 1.8 (SD = 0.95), and 1.85 (SD = 1.04) respectively, where a rating of 1 is very enjoyable. Ratings of AD mean quality ratings for episodes 4, 5, and 8 were 7.6 (SD = 1.79), 7.7 (SD = 1.63), and 7.65 (SD = 1.95) respectively, where a rating of 10 is the highest quality rating. For enjoyment of the show and AD, episode 3 also had positive results (M for show = 1.76, SD = 0.83 and M for description = 1.8, SD = 0.89) (where 1 was very enjoyable and 5 was not enjoyable at all).

The majority of comments in the thematic analysis were for the positive categories in all themes (totaling 41% or 119 of 288 of all comments). In addition to the comments provided in the main body of this paper, S1 Appendix contains a summary of example comments by theme. From the qualitative analysis the AD Style: Positive, and AD General: Positive theme provided some insight into these findings. For the AD Style: Positive theme there were 46 of 288 comments (15.97%). In episode 3, narrated by LC, three participants reported that the narrator’s voice was distinct and theatrical, which contributed to their enjoyment of the show: “I liked the tone of voice; it was clear” and “I found the style more theatrical than the last episode and enjoyed it more.” Two participants indicated their preferences for the narrator’s style: “I liked the narrator’s voice; it was easy to distinguish from the characters in the show,” and “Of the 3 episodes, I have enjoyed this one the most, as I like this narrator more than the one in episode 2.”

In episode 4, narrated by BY, participants who responded were beginning to make comparisons between the narrators, LC and BY. “I think this was a very good style,” and “In episodes 1 and 3, the narrator’s (LC’s) voice seemed to be sped up when there was more to say. That made it hard to understand. This narrator (BY) didn’t do that. I enjoyed listening to him.” In episode 5, narrated by LC, four participants emphasized how much they were enjoying the narrator’s voice, humour, language, and style of description. “Although I don’t like the show, I liked this describer’s voice and language.” One person stated that “the narrator’s comments were starting to go beyond describing the scenes and (were more) entertaining.”

By the last episodes, participants were beginning to distinguish between the style of the description and the show. “I really enjoyed the show because it had a very good style of description.” In episode 8, two participants indicated that the narrator’s style was enjoyable: “This episode is scary but … I enjoyed the narrator’s voice.” One person explained that the describer “sounded friendly and relaxed.”

In the theme of AD–General: Positive, there were 48 of 288 comments (16.66%). In episode 3, narrated by LC, three participants indicated: “description seems more detailed.” “Description was mellow and did not interfere with my enjoyment.” In episode 4, narrated by BY, five participants reported: “The description is contributing to my enjoyment” and “The description provided was much better than previous episodes.” In episode 5, narrated by LC, two participants stated that they enjoyed the description. In episodes 6, 7, & 8, narrated by SC, eleven participants reported: “As I become more familiar with the characters and the storyline, I am finding it all much more enjoyable,” and “I thought the description in episode 8 was superior to the other episodes.”

**Fig 4:**
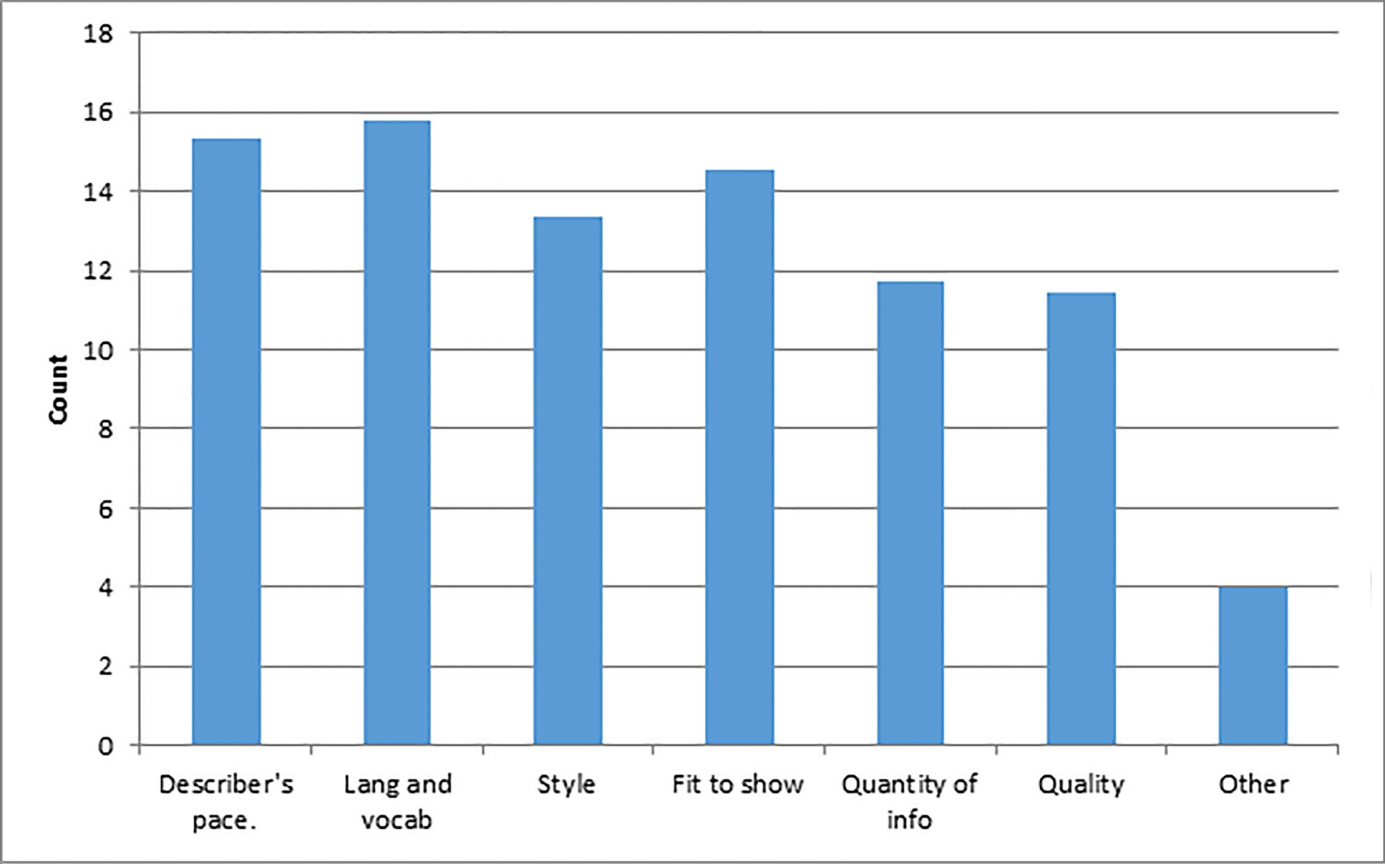
Positive factors.

There were two questions that asked about specific factors that either had a positive (first question) or negative (second question) effect on the ratings of the AD. Fig 4 shows the mean count of each positive factor that was selected. Factors where more than 50% rated them as having the most impact on people’s positive ratings were “describer’s pace” (M = 15, SD = 1.4), “language and vocabulary” (M = 16, SD = 2.7), “fit of description to the show” (M = 15, SD = 1.7) and “style” (M = 13, SD = 2).

In summary, it seemed that participants were beginning to distinguish between the style of the description and the show by becoming more familiar and engaged in the content and the AD by episode 3 [7, 12, 23]. In addition, their understanding of the plot was enhanced as they progressed through the episodes. However, episodes 6 and 7 involved fairly dark humour that some participants found distasteful which may have influenced their ratings of the AD and the show for those two episodes.

Episode 8 was the final episode where participants understood the show, its plot and characters, and it had a positive ending where all the plot elements concluded; Death left the Shuckton. It is not surprising that this episode evoked positive comments. In addition, it seems that the integrated style of description as well as the fit of the description to the show’s style resulted in participant’s positive experience of the show.

### Negative responses

Episodes 1 and 6 consistently scored among the most negative answers. For the question on level of enjoyment of the show, episodes 1 and 6 had means of 2.1 (SD = 0.70) and 2.10 (SD = 1.04) respectively. For enjoyment of the AD, episodes 1 and 6 had means of 2.05 (SD = 0.83) and 2.25 (SD = 091) respectively. For rating of quality episode 1 had a mean of 6.85 (SD = 1.63) and episode 6 had a mean of 6.88 (SD = 1.90).

From the thematic analysis, the negative aspects for all themes comprised 19% of all comments (54 of 288 comments). Twenty-four of 288 (8.33%) negative comments were attributed to the AD Style: Negative theme. Because participants were generally familiar with the non-integrated or conventional style of AD [7, 8, 12, 22], it is not surprising that some of them reacted negatively to the hybrid or integrative style, especially the more theatrical style of LC. Standards styles of AD have restricted the describer to the role of narrator [21, 22] even when there is horrific or hilarious content. In episode 1, narrated by LC, participants contended, “I think the narrator put too much emotion in his tone of voice,” and “I don’t like when the narrator tries to be dramatic or too cute.”

Some participants reported barriers to access pertaining to distinguishing between the voice of the AD narrator and the actors. Two participants had difficulty with distinguishing between the voice of the narrator and the voice of the actors. “At some points it was obvious that the narrator was speaking but at other times, it was hard to tell if the narrator was speaking or a cast member.” This might be due to the complexity of the sound track and the audio mix [2, 21].

In episode 6, narrated by SC, three participants had similar comments: “description style is quite annoying,” and “Editorial comments really take away from my enjoyment of the description.” There were also negative comments for other episodes that were similar but not as frequent. The findings support the literature which affirms that B/LV viewers who were familiar with conventional styles of AD were less likely to prefer alternative styles of AD [7, 8, 18].

In the AD General: negative theme, in episode 1, narrated by LC, two participants stated that they did not like the description. “The description did not seem to give all the information needed for the complete picture of events.” There were no negative general AD comments for episode 6.

**Fig 5:**
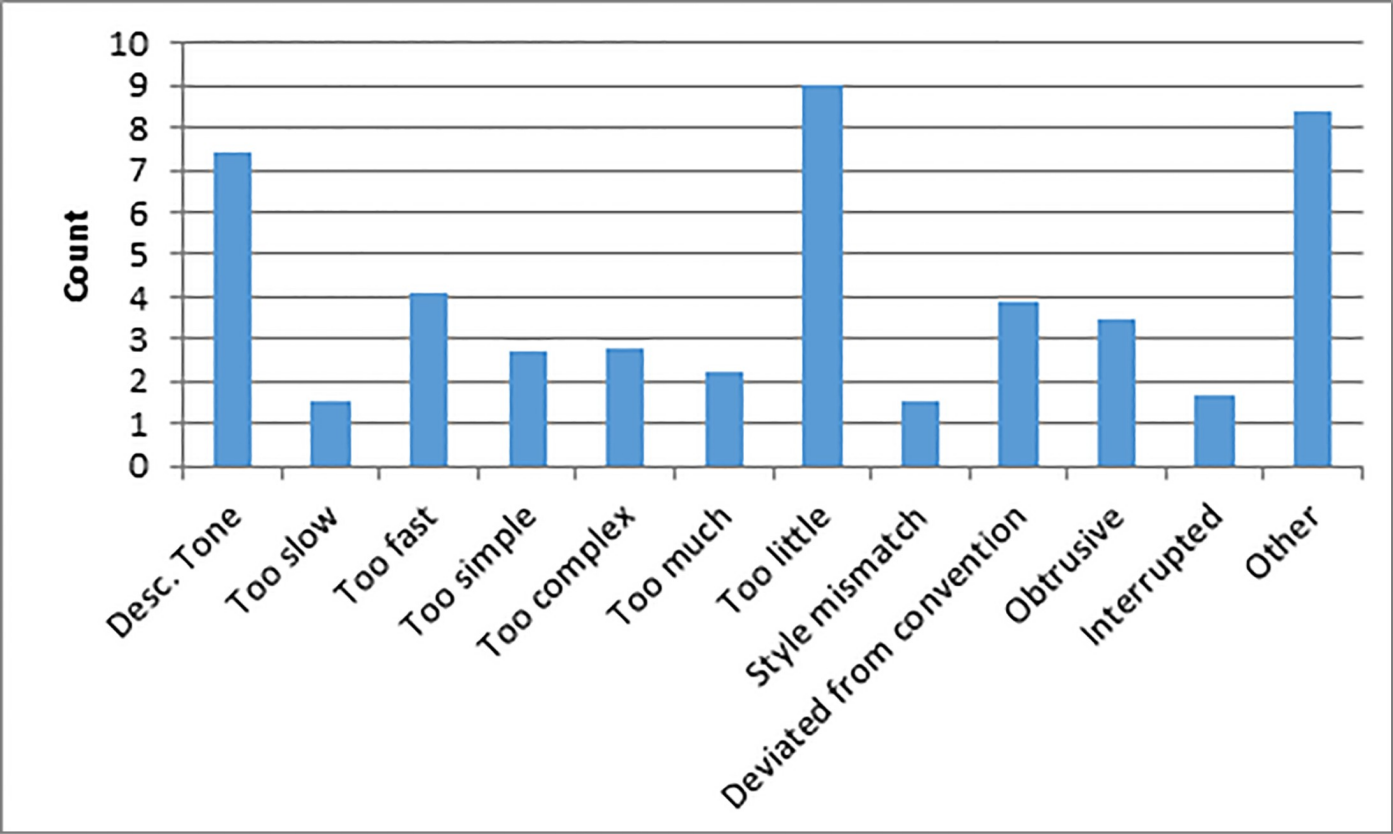
Negative factors.

From the question on the between-episode related to negative factors, Fig 5 shows the mean count of the negative factors that are selected across all episodes. The top two negative factors are: “Describer’s tone of voice” (M = 7, SD = 3.0) and “too little description” (M = 9, SD = 1.7).

The data were combined into per-question responses by describer. Although all describers used the integrated model, three describers were employed during the series. A one-way ANOVA was applied to examine differences between describers, and it showed no differences between describers.

In summary, Episode 1 likely evoked negative comments due to the novelty effect; the AD style was new for most participants and the show was an unusual genre for a majority that took ‘some getting used to.’ The negative reaction to episode 6 was likely caused by the ghoulish subject matter of the episode involving sex with corpses rather than any differences between describers or the describer style. In addition, there was some negative reaction to the integrated style as indicated in the literature. However, it should be noted that the negative comments were less than half of the number of positive comments about the describer’s style and the AD provided.

### Post study questionnaire

Examining the post-study questionnaire, we found that there was a significant Spearman’s rho correlation to a level of p< 0.05 between the responses (N = 24) for “entertainment value of the show” and the “entertainment value of the AD” (r = 0.4), “looking forward to getting each episode” (r = 0.42), the “level of distraction” (r = -0.60) and “level of boredom” (r = -0.67).

There was also a significant Spearman’s rho correlation between responses for the “entertainment value of the AD” and the “level of distraction of the show” (r = -0.56, N = 24, p<0.05). A significant Spearman correlation occurred between the distraction ratings and ratings for whether the description had satisfactory information for the “setting” (r = -0.42, p<0.05) and the “plot” (r = -0.48, p<0.05).

A chi-square analysis was carried out on all questions to determine whether the answers differed from chance. The significant results (p<0.05) are listed in Table 2. Nine of the thirteen questions in the questionnaire had significant chi-square results.

**Table 2:** Chi-square, mean and standard deviation (SD) results for all questions with p< 0.05.

Question	Chi-square (df)	Mean	SD
Entertainment value of the series	9.67 (3)	2.13	1.19
Entertainment value of description	15.58 (4)	2.00	1.10
Fit of description to style of show	11.33 (3)	1.83	0.82
Looking forward to getting each episode	22.00 (3)	1.58	1.02
Distracted by description	10.33 (3)	4.17	1.05
I disliked the series	10.58 (4)	3.67	1.55
I talked to my friends about the show	13.08 (4)	2.42	1.38
Description contained enough information about plot/action	17.67 (3)	1.63	0.92
I was bored with most episodes	17.67 (3)	3.83	1.49

It was found, in the between-episode results, that “level of entertainment” and “enjoyment of the show and AD” was high. Seventy-nine percent of participants (19/24) rated their level of entertainment as “very entertained” or “entertained”, while 5/24 (21%) rated their level of entertainment as “not really entertained” or “not entertained at all”. Regarding the level of entertainment of the AD, 79% were “entertained”, 8% (2/24) were “neutral” and 12% were “not entertained”. Examining the question regarding the fit of the description style to the style of the show, 83% (20/24) of people rated it as “very similar” or “somewhat similar” while 3/24 were “uncertain” and one participant said it was “somewhat dissimilar”. Ninety-two percent (22/24) “looked forward to receiving the next episode”, while 8% (2/24) “did not look forward to receiving the next episode”. Seventy-nine percent (19/24) said that they were “not distracted by the description”, two participants were “neutral” and three participants were “somewhat distracted by the description”. Sixty-two percent (15/24) said that they “liked” the series while 3/24 (12%) were “neutral” and six participants (25%) reported that they “did not like” the series. Finally, 75% of participants were “not bored” with the episodes while 20% said that they “were bored”; one person was “neutral”.

From the AD Style: positive theme, participants’ positive comments about the describers’ styles indicated that tone of voice, humour, language, as well as the fit of description to show, were fundamental to enhancing the entertainment value. There was a division in preference for pace of description, where some participants preferred the faster theatrical style of LC, while others preferred the slower relaxed style of SC.

Fourteen participants also expressed satisfaction with the quality of AD (AD General: Positive theme). Positive comments include: “AD helped me to understand the action when there was no dialogue.” Three participants felt that the AD matched the style of the show. “I liked that the description had the same type of humour as the show,” and “The description was upbeat and matched the comedy.”

From the Show: Positive theme, 25 of 288 (8.68%) occurred. Seventeen participants stated that they enjoyed the show. Comments include: “I love Kids in the Hall,” and “The show was funny.” One person explained, “I really felt that I’m getting to know the various characters in the show. It amazes me that five to seven men can play so many different characters …” In the post survey reflections, one participant asserted, “I found the one-liners really funny and I liked the overall sense of humour. The videos kept me in suspense and the plot was surprising. A lot of characters made me laugh the way they were portrayed.” Some participants reported that the AD influenced their enjoyment of the show. One participant indicated, “My overall enjoyment of the show was not negatively affected by the AD; it had to do with my personal taste. I am sure that without the AD, I would not have enjoyed the show,” “I really enjoyed the show because of the style of the AD,” and “Having to watch the show (with AD) really made me pay attention to what works for me as a viewer.”

From the negative perspective of AD Style, a few participants’ expressions of dislike with the hybrid (integrative) style indicating that their expectation of AD is that it sounds factual, and similar to the newsreader style as affirmed in the literature [1, 2, 7, 18]. They do not seem to regard AD as an art form and a form of entertainment. Such a view is likely a result of the influence of the conventional style of AD that is widely available and accepted by the mainstream culture [8, 18, 21, 22]. However, *Death Comes to Town (described)* is, for most participants, their first exposure to the integrative style approach to AD [18]. Moreover, the show is a dark comedy with a complicated plot. B/LV viewers are faced with having to comprehend the plot as well as listening to a new approach to AD. With no prior experience or exposure to the integrative style, the entertainment value of the narrators’ styles may be overlooked by some participants.

In the post survey, six participants reported negative experiences that fit in the AD General: Negative theme. “I found it (AD) difficult to understand. Describer did not enunciate clearly.” “The describer was inconsistent (during the various episodes). “I felt that I was not getting enough information as I get with audio described movies.” A participant, who watched movies with conventional style of AD, expressed dislike, “I would have preferred more information through the AD.”

Participants who had some previous experience with AD were only familiar with the non-integrated or conventional style [2]. *Death Comes to Town* (described) was their first experience with the hybrid or integrative style. Verbal stimuli are the only stimuli available to most B/LV viewers [7, 12, 18]. Thus, the describers’ voice was regarded as the vehicle for comprehension of visual media [2, 7, 8, 11].

From the AD Show: negative them, 14 of 288 comments (2.77%) occurred. It appears that the negative comments expressed by participants in the study were influenced by their lack of experience with the dark comedy genre as discussed in the literature [7, 12, 18, 23]. Their comments include “This is not my kind of show. The satire was crude; the comments shallow.” “I found the show a bit dull. “This type of video is not something I would choose to watch. Sex with a corpse is ghoulish, although I did like a lot of the humour.” “I do like dark humour, but I am not enjoying the show as it did not fulfill my expectations. I even found it boring.” Five participants reported problems with comprehending the dialogue and distinguishing specific characters. “I found it difficult to follow the plot because the cast all have similar voices.” “The scenes were moving too fast. It was hard to tell who was speaking.”

In summary, even though participants may have a preference for a particular style of a narrator, they recognized the contribution of AD to their entertainment experience. e.g. “It (AD) was clear and did not interfere with the dialogue.” “I loved the description … I hope that someday that (AD) will be built in.” It seems that enjoyment of the show and enjoyment of the AD are inter-dependent. The majority of participants expressed satisfaction with the AD. This may be due to their enjoyment of the humour, satire and suspense of the mellow-drama. They also expressed feelings of engagement with the show. As one viewer put it, “Having to watch the show (with AD) really made me pay attention.” It is evident from their positive comments that the show introduced B/LV viewers to a more complex and Avant–Garde style of comedy.

### Plot/action, character, setting

Although the majority of participants enjoyed the show, the complexity of the plot and the dark humour impeded the entertainment value for some participants. As several participants affirmed, the AD was salient to their comprehension and enjoyment of the show. However, the rapid pace of the show warranted an equally rapid AD style. Some participants were distracted by the pace and style of the show with AD. The entertainment value of the show was heavily dependent on the response to visual stimuli, which is problematic for B/LV viewers. Due to the limited space and time permitted to insert AD, it cannot fully compensate for visual stimuli.

Walczak and Fryer [7] argues that “AD has the potential of triggering emotional reactions in both sighted and B/VIP audiences …” (p. 8). Thus, the negative reactions to *Death Comes to Town (described)* as reported by some participants are not unusual [8, 10, 19].

Negatives reaction to AD may be influenced by type of genre and individual preferences for particular types of shows [10, 12, 19, 36]. The dark comedic style of *Death Comes to Town* is complex and Avant–Garde and may be regarded as unappealing. Moreover, negative reactions to the AD may have been influenced by participant’s discomfort with the show itself [2, 8, 10, 19].

Examining the distribution of responses for the level of descriptive detail for the plot, characters, and settings show that for information about the plot, 87% “agreed” that the description provided enough information to understand the show, while one person was “neutral” and two people “somewhat disagreed”. Sixty-four percent of participants “agreed” that more information was required about the characters, 12% were “neutral” and 21% “disagreed” that more information was required. Finally, 59% “agreed” that there was sufficient information about the setting, while 12% were “neutral” and 28% “wanted additional information”.

The episodes were analysed to examine the distribution of plot (action), character, and setting descriptions. The majority of description (67% of words) was related to plot description and action words, such as “Suddenly they all spin away holding the baby dolls.” Character-oriented descriptions (facial expressions, clothing, and posture) consisted of 19% of words. Setting identification descriptions such as “the seashore” comprised 13% of the descriptions and, the final 1% were setting descriptions that contained descriptive elements beyond the name (e.g., “the front steps facing the court”).

Full comprehension of the plot enhances the entertainment value for all viewers [7, 23, 36]. Thus, it is essential for B/LV viewers to understand and be engaged in the plot. In this series, however, there were relatively few comments about wanting more plot details. The More action details theme had 2.08% of the comments (6 of 288). In all eight episodes, six participants reported that they would have liked more AD on the plot and action. They contended that “some actions were not described,” and “My sighted wife told me that some actions were not described.” One participant argued that even when actions were described, there wasn’t enough detail for her to follow the plot. “He (describer) assumed that we understood some things such as ‘the devil self-chiropractic;’ I did not know what (was being done).” The same comments were restated in the post survey. This suggests that participants were generally satisfied with the amount of action detail provided and this adheres to findings from [40]. In order to provide more action description would require compromising descriptions of other elements or having an extended description option where viewers could find more information online or as extra information on DVD. Participants further affirmed that familiarity with the plot and the style of the different describers’ respective styles enhanced the entertainment value.

As emphasized in the AD literature [2, 7, 10, 23, 41], there is limited time and space to include extensive information on plot especially a multi-leveled plot as that of *Death Comes To Town*. Lopez, Kearney and Hofstadter [21], asserted that B/LV viewers will have to rely on other access strategies such as digital media, which would allow them to “personalized their access strategies” to obtain additional information that is not provided by AD (p. 4).

From the thematic analysis, the theme of More character detail had 24 of 288 comments (8.33%) of the total comments. In this theme, participants reported that they desired more character details. However, the complexity *of Death Comes to Town* and the limited space and time for inserting AD, prohibited extensive description of each character [12, 18]. In episodes 1, 2, & 5, seven participants desired more character description. Participants contended that they were often confused, as they were not aware that the characters were all men in drag. “The description doesn’t state that the characters were men in drag.” “I could not tell who were girls in the movie–voices were high.” Three participants stated that they would have liked to have known what clothing each character was wearing.

In episode 3, three participants indicated that they would like more information about the facial expressions of the actors. “There was no information about people’s facial expressions.” In episodes 4, 6, 7, & 8, one participant asserted: “Describing the characters more fully and the change of scenery would have added to my enjoyment.” Eight participants made similar comments in the post survey. “I felt that the description was lacking–overall character description.” Fryer and Freeman [8] suggested that facial expressions makes B/LV viewers “feel a part of it” (p. 9) and that B/LV want more than just the plot.

Similar to other researchers [8, 10, 18, 19, 23], the findings for setting show that it is also salient to B/LV viewers’ comprehension and engagement. The More setting details theme had 5.2% of the comments (15 of 288). In all eight episodes, 14 participants affirmed that more description on settings would have contributed to their enjoyment of the show. Examples provided include: houses, location of events, the judge’s gavel, the vet’s office, the mayor’s office, the bar, and the bar attenders. One participant argued that setting description was often omitted. “There were many parts of the show where I had to ask what was happening because there was no description.” Another participant asserted “More verbal description such as clothing, type of weather, danger such as mom and son on the train track could have been helpful.” Similar comments were reported in the post survey. One participant elaborated, “In the case of the cat at the vet’s, I was not sure if the cat was really sick or if they were trying to cheat the owner of his money. I was often left wondering what was going on, especially when the describer made comments like, ‘that must hurt’.”

Finally, there were 26 of 288 comments (9.02%) related to not having enough details. In all eight episodes, five participants stressed their desire for the provision of more detailed information in the AD: “Description didn’t seem to give all the information for a complete picture” and “I could have used more elaborate description.” One participant expressed frustration: “I would have liked more information.” Six participants gave specific examples of the type of details. “Describe the room and who was there and what they were doing” and “Narrator did not describe what someone was holding in their hand that were being given to the trans-genders or Ricky going into the car.” Three participants proposed that a preview of background information and the cast would be helpful. “Some preview description is needed before the show starts,” and “Not enough information about the background, the colours, clothing, the setting and the action.”

Television and film are considered visual media as they are heavily dependent on visual stimuli. It is therefore extremely challenging to create a comparable entertaining experience through audio stimuli [2, 7, 19, 21, 42]. A further challenge is the limited space and time available to insert AD within the secondary audio track. Describers and script-writers are forced to make difficult choices [2, 7, 12, 18, 36] and compromises. Unlike other genres such as documentaries and drama, where the sensory elements are usually complementary, enjoyment of comedy relies on trivial details and subtleties to enhance entertainment value [8, 12, 41]. Aspevig and Pedersen [18] contended that one of the barriers to making humour fully accessible to B/LV viewers is the limited time provided to describe the subtleties. These findings are supported by Lopez, Kearney and Hofstädter [21], which show that B/LV viewers may require multiple access strategies as well as “personalized strategies” to fully engage in visual media.

Gagnon et al., [41] provided a typology of AD based on 11 different shows of varying lengths and genres. They found that action descriptions comprised the majority of description words, followed by character descriptions and setting/décor. In our study, viewer ratings indicated that the information contained in the description about the plot was sufficient; however, more description for the characters seemed desirable. Comments such as “I would have liked more description of settings, characters, etc.” were common.

It is indeed a challenge to produce AD that incorporates extensive detailed descriptions of characters, settings, and action/plot. As discussed in Fels and Naraine [12], “Given the limited time and space available for inserting description in a television show, directors, describers, and producers have to consider what information is essential pertaining to character, settings, and action/plot to contribute to the entertainment value of the show” (p. 4). As noted in [12], the optimum level of each description type can only be determined by further research. A major challenge is determining how much of each type of description will be regarded by B/LV viewers as sufficient. Given the comments made by participants in this study, it seems that viewers who are congenitally blind (eleven participants) were not as concerned with setting description as viewers who are adventitiously blind (thirteen participants). It also seems that B/LV viewers’ desire for extended AD and extra content information was not specific to *Death Comes to Town*, but extends to all genres. A possible future development is to find ways to include extended AD content that can be consumed as an “extra” content existing in a separate venue (e.g., Web site) or within the show itself, where users can pause the show and listen to the extra AD content.

### Pace

A rough estimate for the pace for each describer was calculated by dividing the number of all words in an audio description segment by the number of seconds for each describer. There was no significant difference between describers based on a one-way ANOVA (Levine statistic was not significant); [F(2, 151) = 2.08, p>0.05]. LC had a mean description rate of 3.81 words per second (wps), SD = 1.18, BY a mean of 3.28 wps, SD = 1.01, and SC a mean of 3.46 wps, SD = 1.29. However, for all three describers there were within describer variations that related to the number of seconds available. However, the maximum and minimum wps varied considerably particularly for the shorter time intervals. For example, BY had one 7 wps for a two-second interval and 2 wps in three one-second intervals. The 7 wps in the two second interval (e.g., 14 words spoken in two seconds) likely sounded very fast paced. Each describer had this level of variability in their descriptions. While this is only a very rough estimate and does not account for different word sizes, pronunciation, and cadence, it allows some comparison between describers.

Some insight about the impact of pace on audience preferences was provided in the subjective comments. For Pace; Positive theme there were 15 of 288 comments (5.20%). Eight participants reported that the pace was satisfactory. In episode 2, narrated by BY, three participants reported that they were pleased with the pace where BY has the slowest pace at 3.28 wps, SD = 1.01. “Description tone and pace was much better than episode 1.” In episodes 3 & 4, narrated by LC and BY respectively, one participant indicated a preference for a faster pace. LC had the fastest pace at 3.81 wps and SC had a pace between LC and BY at 3.46 wps. “I am convinced that a really fast pace narrator could give more information and the viewers would understand the details.” Two participants argued that the narrator’s pace was suitable. “The narrator’s pace was very good.” In episodes 7 & 8, narrated by SC, two participants reported that the pace was adequate. “The pace, pitch and amount of information were amazing.” In the post survey, six participants commented on the pace: “I liked the pace of the description in relation to the show because it did not interfere with the dialogue.”

In the Pace: Negative theme there were 14 of 288 comments (4.86%). In all eight episodes, some participants contended that the pace was too fast. In episode 1, narrated by LC, five participants reported that the pace was too fast. “The description was too fast in the beginning some of the words could not be (heard).” LC did have the fastest pace but it was not significantly different from the other two. In addition, there were one instance where the pace was 6 wps and two instances where it was at 5 wps where all three instances were during one and two second time intervals. Longer time intervals had a slower pace. In episode 2, narrated by BY, three participants contended that the AD was fast. However, the mean pace for BY was slowest of the describers although there was one instance where the pace was 7 wps and two at 5 wps (all three instances delivered during short duration time intervals of two seconds or lower). Moreover, the narrator’s voice was not distinct from the voices of some of the actors, which resulted in distraction. “It was hard to tell who was speaking.” In episodes 3 & 5, narrated by LC, two participants reported, “The narrator’s speed was too fast.” Two participants reported that the pace was too fast in episode 7 narrated by SC. SC’s pace was between BY and LC but in episode 7 there were three instances where the pace was 6, 7, and 8 wps (all in time intervals of less than two seconds). In the post survey, three participants reported that the pace was too fast overall.

The AD for *Death Comes to Town* was produced using the integrated method, which entailed inserting the AD post-production. This means that the space available for description was limited. It would also seem that the shorter time intervals tended to have faster descriptions which is not surprising. As there was limited time and space to insert AD between the dialogue, it was expected that some participants would find the pace of the AD too fast, particularly for the shorter times. Also, participants who reported that the pace was satisfactory appeared to be more comfortable and engaged with the integrative style. These findings support previous research studies [2, 7, 8, 23] on AD, which indicate that enjoyment of AD style and pace is impacted by engagement and comfort levels with the show. Additional research is required to determine pace preferences and limits for a variety of time intervals. However, the majority of participants found the pace satisfactory and in keeping with the specific style of this dark comedy. In addition, it seems that participants have different preferences for pace. Having the opportunity to speed up or slow down description pace (containing more or less content respectively) may be a solution to satisfying this need. A “one-size-fits-all” should be reconsidered in favour of a user-controllable pace, similar to how pace can be controlled for sign language videos [2, 8, 10, 19, 35].

### Language level

A Flesch-Kincaid Grade level analysis [43] was also carried out on the audio description scripts for all describers, and each was assessed to be at a college graduate level. From the during and post-study questionnaires the Language theme contain 7 of 288 comments (2.43%) for episodes 1, 4, 6, 7, 8 and the post survey. As a dark comedy, *Death Comes to Town* portrayed various elements of comedy, including vulgarity, irony, parody, and perverse humour. Thus, the diction used by the AD narrators reflected such elements. It is not surprising that some participants expressed dislike with the diction used in the AD. “I do not think words like ‘ass’ should be used” (episode 1). “…saying that Larry landed in his grave ‘ass up’ could have been said differently” (episode 7). The discomfort expressed by participants seemed to result from a lack of experience with dark comedy and the integrative style of AD.

Two participants contended that the diction in episode 4 was ambiguous. “Make language clear. Don’t say, ‘he is smoking a fatty.’ I don’t know what that is. Say he is smoking marijuana.’ ‘Don’t call breasts ‘women’s lump’ I want to know exactly what is going on just like sighted people.” The desire for precise language rather than diction that portrays the style of the comedy suggests that some participants are influenced by the conventional/newsreader style of AD. It seems that some participants who found the language offensive may not be as familiar with elements of dark comedy. There were no comments that the language level was too complex or advanced.

In the integrative style of AD, the use of language and diction is salient to ensuring that the style of AD matches the style of the show and type of genre. Szarkowska [24] explains that “Auteur description differs from conventional AD in the way the who, what, where, and how of the film are described. Departing from the notion of objective description, it eagerly embraces vivid and emotional language” (p. 383).

As a dark comedy, *Death Comes to Town* portrayed various elements of comedy, including vulgarity, irony, parody, and perverse humour. Thus, the diction used by the AD narrators reflected such elements.

A final thematic category was Technical issues and comprised 8 of 288 comment (2.77%). Technical considerations were not an important factor in this study. However, in episodes 1, 5, & 8, eight participants reported problems such as background noise, variations in volume levels for the AD, as well as the volume of the dialogue. “Description was difficult to hear clearly at times” and “Background noise sometimes interrupted the AD.” This impeded the entertainment value for some participants but it is likely due to problems with the hardware (speakers, headphones and local volume levels) that participants used to listen to the show and not mixed volume levels of the AD and original audio track, and/or hearing abilities of the participants. Sound quality and other technical issues could not be evaluated as participants watched the shows using their own television and assistive technology devices. Future research should provide hardware guidelines as well as participants to describe their listening devices as well as measure hearing levels in order to better determine the source of noise or volume levels.

## Limitations

There were numerous limitations in this research, including having a low number of participants. However, the repeated and longitudinal nature of the research provided a unique opportunity to explore audio description for an entire series, whereas most prior research uses only a single exposure to any one piece of content. In addition, there was an unequal number of episodes described by each describer, which may have affected novelty and familiarisation factors. Future work with longitudinal data should examine the impact of describer approach on novelty and change in viewer opinions over time. One of the difficulties with basing research conclusions on one-off exposure to AD is that participants may be reacting to the novelty or unfamiliarity of the content and AD approach. Another limitation is that only one genre was examined; longitudinal reactions to other genres may be different. In addition, describers may want to take different AD approaches than were taken in this research, which will influence audience reactions. The words per second calculation for pace is a very course grain assessment of speaking rate. A different grain size, such as phonemes or syllables, or a different variable altogether could be considered for a more accurate or descriptive assessment. However, this was beyond the scope of this paper and could be examined in future research. Finally, while some of the methodology used has been used in other studies, these were single episode studies and as a result it has not been subjected to validation. Future research could undertake the necessary steps towards validation.

## Conclusion

In summary, *Death Comes to Town (described)* was the first integrative AD for a TV show experienced by the B/LV participants in this study. This type of dark comedy was dependent on visual stimuli for its entertainment value. Given that B/LV viewers must rely mainly on verbal stimuli, the describer’s tone of voice, style, humour, and language were salient to the comprehension and enjoyment of the AD. Initially, participants seemed to be overwhelmed by the complicated plot and a new integrative approach to AD but as the show progressed they were able to distinguish between their enjoyment of the AD versus the show–for some they liked the AD regardless of the show. For the entire 8 episodes of *Death Comes to Town* with different styles and describers over the series, participants reported that they were entertained by the AD. Participants further affirmed that familiarity with the plot, pace, the style of the different describers’ respective styles, and fit of the description of the show enhanced the entertainment value of the show.

The majority of participants responded positively to the all of the describers’ styles. Although there were no significant differences between describers for enjoyment, some preferences for one of the three describers were expressed. These mostly related to pace or emotional quality. Participants also expressed a desire for more detailed information, and extensive description pertaining to plot, character, and setting descriptions. However, it was not possible to incorporate description of all visual stimuli within the space available, and thus, extended descriptions, where the show can be paused to allow for more detailed description was recommended as one solution to this dilemma. Future work could examine the impact of having a pause/extended description feature on the entertainment and enjoyment of a show as well as examine the longitudinal impact of various factors such as describer style for other genres and series lengths.

## Supporting information

S1 Dataset Dataset. (ZIP)

S1 Appendix (DOCX)

S2 Appendix (DOCX)

## References

[1] Snyder J. Audio description: The visual made verbal. Int Congr Ser. 2005;1287(Journal Article):935–9.

[2] Udo JP, Fels DI. The rogue poster-children of universal design: Closed captioning and audio description. J Eng Des [Internet]. 2009;(Journal Article). Available from: http://www.informaworld.com/smpp/content~db=all~content=a916629020

[3] Sourbati M. Disabling communications? A capabilities perspective on media access, social inclusion and communication policy. Media Cult Soc. 2012;34(5):571–587.

[4] Bachmeier C. Barrier-free access to audiovisual content–a fundamental human right. IRIS Plus. 2014;3.

[5] Ellis G. Impairment and Disability: Challenging Concepts of ‘Normality.’ In: Researching Audio Description. Springer; 2016 p. 35–45.

[6] Alper M. Promoting emerging new media literacies among young children with blindness and visual impairments. Digit Cult Eduction [Internet]. 2015 [cited 2018 Sep 1];4(3). Available from: http://www.digitalcultureandeducation.com/uncategorized/dce1077_allen_2012_html-2/

[7] Walczak A, Fryer L. Creative description: The impact of audio description style on presence in visually impaired audiences. Br J Vis Impair. 2017;35(1):6–17.

[8] Fryer L, Freeman J. Cinematic language and the description of film: Keeping AD users in the frame. Perspectives (Montclair). 2013;21(3):412–426.

[9] Sueroj K, Sarakornborrirak P. An Overview of Audio Description on Thai Television. In: Researching Audio Description. Springer; 2016 p. 205–224.

[10] Vladica F. Understanding entertainment value: An investigation into the subjectivity of people who experience entertainment. Unpubl Dr Diss Tor Can Ryerson Univ. 2012;

[11] Whitfield M, Fels DI. Inclusive design, audio description and diversity of theatre experiences. Des J. 2013;16(2):219–238.

[12] Fels DI, Naraine M. When audio description came to town: a longitudinal study. FICCDAT Tor June. 2011;

[13] Government of Canada CR and TC (CRTC). TV Access for People with Visual Impairments: Described Video and Audio Description [Internet]. 2008 [cited 2018 Apr 10]. Available from: https://crtc.gc.ca/eng/info_sht/b322.htm

[14] Winseck D. Netscapes of power: Convergence, consolidation and power in the Canadian mediascape. Media Cult Soc. 2002;24(6):795–819.

[15] Newman L, Browne-Yung K, Raghavendra P, Wood D, Grace E. Applying a critical approach to investigate barriers to digital inclusion and online social networking among young people with disabilities. Inf Syst J. 2017;27(5):559–588.

[16] Weigand M, Zylka J, Müller W. Media competencies in the context of visually impaired people. In: European Conference on Information Literacy. Springer; 2013. p. 190–197.

[17] Alper M. Developmentally appropriate new media literacies: Supporting cultural competencies and social skills in early childhood education. J Early Child Lit. 2013;13(2):175–196.

[18] Aspevig K, Pedersen I. Death Comes to Town, irreverent humour, and accessibility for the blind and low-visioned. Can J Commun. 2011;36(3).

[19] Ramos M. The emotional experience of films: does Audio Description make a difference? The Translator. 2015;21(1):68–94.

[20] Caro MR. Testing audio narration: the emotional impact of language in audio description. Perspectives (Montclair). 2016;24(4):606–634.

[21] Lopez M, Kearney G, Hofstädter K. Audio Description in the UK: What works, what doesn’t, and understanding the need for personalising access. Br J Vis Impair. 2018;0264619618794750.

[22] Ofcom. Ofcom’s code on television access services. 2015 May p. 1–23.

[23] Orero P. Sampling audio description in Europe. Media Subtitling Deaf Audio Descr Sign Lang. 2007;111–125.

[24] Szarkowska A. Auteur Description: From the Director’s Creative Vision to Audio Description. J Vis Impair Blind. 2013;107(5):383–387.

[25] Bordwell D, Thompson K. Film art: An introduction Vol. 6th. New York: McGraw Hill; 2001.

[26] Weston J. Film Director’s Intuition Michael Wiese Productions; 2003. (Michael Wisese Productions).

[27] Rea P., Irving D.K. Producing and directing the short film and video. Focal Press; 453 p.

[28] Smith J., Bordwell D., Thompson K. Film Art: An Introduction, 11th ed New York: McGraw Hill; 2016. 544 p.

[29] Musburger R.B., Kindem G. Introduction to media production: the path to digital media production. Focal Press; 2012. 376 p.

[30] Kozloff S. Overhearing film dialogue University of California Press; 2000. 327 p.

[31] Raney A.A. The Psychology of Disposition-based Theories of Media Enjoyment In: Psychology of entertainment. New York: Routledge; 2006 p. 137–50.

[32] Romero-Fresco P. Accessible filmmaking: Joining the dots between audiovisual translation, accessibility and filmmaking. J Spec Transl. 2013;20:201–223.

[33] Xue Fei. Audio description in the Chinese community [Internet]. [Toronto]: Ryerson University; 2011 [cited 2018 Sep 1]. Available from: http://digital.library.ryerson.ca/islandora/object/RULA%3A2946

[34] Bergson H. Laughter: An Essay on the Meaning of the Comic. New York: Macmillan; 1911.

[35] Maxwell N. Black Comedy and the Principles of Screenwriting: the Actions [Internet]. [Melbourne]: RMIT University; 2011 [cited 2018 Sep 1]. Available from: http://researchbank.rmit.edu.au/eserv/rmit:7859/Maxwell.pdf

[36] Fels DI, Udo JP, Diamond JE, I DJ. A comparison of alternative narrative approaches to video description for animated comedy. J Vis Impair Blind. 2006;100(5):295–305.

[37] Udo JP, Fels DI. Enhancing the entertainment experience of blind and low-vision theatre-goers through touch tours. Disabil Soc. 2010;25(2):231–40.

[38] Udo JP, Fels DI. Suit the action to the word, the word to the action”: An unconventional approach to describing Shakespeare’s Hamlet. J Vis Impair Blind. 2009;103(3):178–84.

[39] Udo JP, Fels DI. Re-fashioning fashion: an exploratory study of a live audio-described fashion show. Univers Access Inf Soc. 2010;9(1):63–75.

[40] Fels DI, Udo JP, Ting P, Diamond JE, Diamond JI. Odd Job Jack described: a universal design approach to described video. Univers Access Inf Soc. 2006;5(1):73–81.

[41] Gagnon L, Laliberte F, Lalonda M, Beaulieu M. Toward an Application of Content-Based Video Indexing to Computer- Assisted Descriptive Video. In 2006. p. 8.

[42] Fels DI, Udo JP, Diamond JE, Diamond JI. A Comparison of Alternative Narrative Approaches to Video Description for Animated Comedy. J Vis Impair Blind. 2006;100(5):295–305.

[43] Kincaid J. P., Fishburne R. P., Rogers R. L., & Chissom B. S. Flesch-Kincaid Grade Level Memphis: United States Navy; 1975.

