# Supplementary material for: Impacts on quality: Enjoyment factors in blind and low vision audience entertainment ratings: A qualitative study
Source: PLoS One. 2018 Dec 3;13(12):e0208165. doi: 10.1371/journal.pone.0208165 (PMC6277089; doi:10.1371/journal.pone.0208165)
Supplement: S1 Appendix — (DOCX) [file pone.0208165.s002.docx]

# Appendix A: Table of example comments for each theme by episode and describer

| **Episode (Describer)** | **Theme/ Category** | **Positive Comments** | **Negative Comments** |
| --- | --- | --- | --- |
| Episode 1 (LC) | *Style of Narrator* | “I liked the describer’s voice and humour." “Description matched what was happening” | “I think the narrator put too much emotion in his tone of voice”. "... it was hard to tell if the narrator was speaking or a cast member.” |
| Episode 2 (BY) |  | “I enjoyed it because I am familiar with the plot and the way the narrator describes.” | “Even after watching the show once before, I still find it hard to differentiate between the different voices of the cast and the narrator." |
| Episode 3 (LC) |  | “I liked the narrator’s voice it was easy to distinguish from the characters in the show”. | “Things should be described exactly as they are instead of being interpreted. I would like to make this interpretation myself.” |
| Episode 4 (BY) |  | “I think this was a very good style.” "I enjoyed listening to him. ” | “The description should be done more formally – a simple play by play.” |
| Episode 5 (LC) |  | “Although I don’t like the show, I liked this describer’s voice and language.” | “The description is like a sport commentary style.” "Narration “intrusive” at times, but worked well when Death was doing a dance. |
| Episode 6 (SC) |  | “there was a noticeable difference in style”. | “Editorial comments really take away from my enjoyment of the description.” |
| Episode 7 (SC) |  | “best description so far”. | No Comments |
| Episodes 7 & 8 (SC) |  | no comments | Description needs to be more factual. I.e. say character is ‘smiling’ rather than ‘happy’.” |

| **Episode (Describer)** | **Theme** | **Positive Comments** | **Negative Comments** |
| --- | --- | --- | --- |
|  | *AD General* |  |  |
| Episode 1 (LC) |  | “The AD facilitated my understanding of what was going on.” | “The description did not seem to give all the information needed for the complete picture of events.” |
| Episode 2 (BY) |  | “I like it when the sounds of the description stand out from the show.” | “I find that it (AD) doesn’t always describe when I think it is necessary”. |
| Episode 3 (LC) |  | “Description was mellow and did not interfere with my enjoyment.” | “There was (sic) times when the action was not described -- I didn’t understand (the show).” |
| Episode 4 (BY) |  | “The description is contributing to my enjoyment.” | “it is difficult when you don’t like the show to like any of the audio description.” |
| Episode 5 (LC) |  | Enjoyed the description. | “the description was (distracting) ... it was way too intrusive at times.” |
| Episodes 6, 7 & 8 (SC) |  | “As I become more familiar with the characters and the storyline, I am finding it all much more enjoyable.” "description in episode 8 was superior to the other episodes.” | AD was, “inadequate” and “incomplete.” |

| **Episode (Describer)** | **Theme** | **Positive Comments** | **Negative Comments** |
| --- | --- | --- | --- |
|  | *Pace of AD* |  |  |
| Episode 1 (LC) |  | Pace was satisfactory. | “The description was too fast in the beginning.” |
| Episode 2 (BY) |  | “Description tone and pace was much better than episode 1.” | AD was fast. “It was hard to tell who was speaking.” |
| Episodes 3 & 5 (LC) |  | no comments | “The narrator’s speed was too fast.” |
| Episodes 3 & 4 (LC & BY) |  | “I am convinced that a really fast pace narrator could give more information and the viewers would understand the details.” | no comments |
| Episode 6 (SC) |  | no comments | no comments |
| Episodes 7 & 8 (SC) |  | “The pace, pitch and amount of information were amazing.” | "The pace was too fast in episode 7." |

| **Episode (Describer)** | **Theme** | **Positive Comments** | **Negative Comments** |
| --- | --- | --- | --- |
|  | *More Character Details* |  |  |
| Episodes 1, 2 & 5 (LC) & (BY) |  | No comments | “The description doesn’t state that the characters were men in drag.” |
| Episode 3 (LC) |  | No Comments | “There was no information about people’s facial expressions.” |
| Episodes 4, 6, 7 & 8 (BY) & (SC) |  | No comments | “Describing the characters more fully and the change of scenery would have added to my enjoyment.” |
|  | *More Setting Details* |  |  |
| Episodes 1 to 8 (LC), (BY( & (SC) |  | no comments | More discription on setting would have contributed to enjoyment of show. |
|  |  | no comments | “There were many parts of the show where I had to ask what was happening because there was no description.” |
|  |  | no comments | “More verbal description such as clothing, type of weather ... could have been helpful.” |
|  |  | no comments | "I was often left wondering what was going on, especially when the describer made comments like, ‘that must hurt’.” |
|  |  |  |  |

| **Episode (Describer)** | **Theme** | **Positive Comments** | **Negative Comments** | |
| --- | --- | --- | --- | --- |
|  | *More Action Details* |  |  | |
| Episodes 1 to 8 (LC), (BY) & (SC) |  | No Comments | More AD on plot and action. “some actions were not described.” “My sighted wife told me that some actions were not described.” | |
|  |  | No Comments | Even when actions were described, there wasn't enough details to follwow the plot. | |
|  |  | No Comments | “He (describer) assumed that we understood some things such as ‘the devil self-chiropractic;’ I did not know what (was being done).” | |
|  |  | No Comments | “The cat needed to be described fully to understand what was going on.” | |
|  | *Not Enough Details General* |  |  | |
| Episodes 1 to 8 (LC), (BY) & SC) |  | No Comments | “Description didn’t seem to give all the information for a complete picture.” | |
|  |  | No Comments | Examples of details desired: “Describe the room and who was there and what they were doing.” | |
|  |  | No Comments | “Some preview description is needed before the show starts.” | |
| **Episode (Describer)** | **Theme** | **Positive Comments** | | **Negative Comments** |
|  | *Language* |  | |  |
| Episodes 1, 4, 6, 7 & 8 (LC(), (BY) & (SC) |  | No Comments | | “I do not think words like ‘ass’ should be used” (episode 1). |
|  |  | No Comments | | “Make language clear. Don’t say, ‘he is smoking a fatty.’ |
|  | *Show* |  | |  |
| Episodes 1 to 8 (LC), (BY) & (SC) |  | “I love Kids in the Hall.” “Schucktown is Hilarious!” | | “This is not my kind of show. The satire was crude; the comments shallow.” |
|  |  | “I really felt that I’m getting to know the various characters in the show. It amazes me that five to seven men can play so many different characters...” | | “This type of video is not something I would choose to watch. Sex with a corpse is ghoulish, although I did like a lot of the humour.” |
|  |  | "The videos kept me in suspense and the plot was surprising. A lot of characters made me laugh the way they were portrayed.” | | “I do like dark humour, but I am not enjoying the show as it did not fulfill my expectations. I even found it boring.” |
|  |  | “My overall enjoyment of the show was not negatively affected by the AD; it had to do with my personal taste. I am sure that without the AD, I would not have enjoyed the show.” | | “I found it difficult to follow the plot because the cast all have similar voices.” |
|  |  | “I really enjoyed the show because of the style of the AD.” | | “The scenes were moving too fast. It was hard to tell who was speaking.” |
