# Supplementary material for: Impacts on quality: Enjoyment factors in blind and low vision audience entertainment ratings: A qualitative study
Source: PLoS One. 2018 Dec 3;13(12):e0208165. doi: 10.1371/journal.pone.0208165 (PMC6277089; doi:10.1371/journal.pone.0208165)
Supplement: S2 Appendix — (DOCX) [file pone.0208165.s003.docx]

# Appendix B: Pre, Post and Mid questionnaires

Note: all questionnaires were completed electronically as either a word document or as submission through an online survey design tool.

**Pre-Questionnaire: Death Comes To Town**

We would like to understand a bit about you and your opinion of audio description before and after each episode of watching Death Comes to Town. Thanks in advance for helping us with this project. We hope you enjoy the show.

1. What is your age?

- Under 18
- 19 to 29
- 30 to 39
- 40 to 49
- 50 to 59
- 60 and over

1. What is your level of vision impairment?
   - Blind
   - Low-vision
   - Sighted
2. What is the highest level of education that you have completed?
   - Elementary School
   - High School
   - College
   - Undergraduate Degree
   - Masters Degree
   - Doctorate
3. How many hour of television do you watch per week?
   - I do not watch television
   - Less than 2 hours
   - 2- 5 hours
   - 5-10 hours
   - 10-15 hours
   - 15-20 hours
   - More than 20 hours
4. Please rate your level of familiarity with audio description
   - Very familiar
   - Familiar
   - Somewhat familiar
   - Not very familiar
   - Not familiar at all
5. When audio description is available, how often do you use it?
   - I do not watch television
   - Never watch television with audio description
   - Sometimes watch television with audio description
   - Frequently watch television with audio description.
6. What quality would you rate current audio description available for television

- Very poor
- Poor
- Don’t care
- Good
- Very good
- I do not watch television

1. How often do you watch television with other people?
   - I do not watch television
   - Never watch television with others
   - Sometimes watch television with others
   - Frequently watch television with others
2. When you watch television with others, how often do you request their assistance in understanding what is occurring on screen?
   - I do not watch television
   - Frequently ask for assistance
   - Sometimes ask for assistance
   - Never ask for assistance
3. If you were to assess a show’s audio description, what factors would you consider important in rating the quality of the show?
4. What do you expect from the television show “Death Comes To Town”?
5. What do you expect from the audio description of Death Comes to Town?

Mid Questionnaire: Death Comes to Town

You have watched XX of YY episodes of “Death Comes To Town”, a miniseries by The Kids In The Hall. Please tell us your thoughts on the show and its description thus far.

1. Please rate your level of enjoyment of this XX episode of the show
   - Enjoyed the show very much
   - Somewhat enjoyed the show
   - Uncertain
   - Not really enjoying the show.
   - Not enjoying the show at all
2. Please rate your level of enjoyment of the audio description of this episodes of the show
   1. Enjoyed the show very much
   2. Somewhat enjoyed the show
   3. Uncertain
   4. Not really enjoying the show.
   5. Not enjoying the show at all
3. Out of ten, please rate the quality of the audio description of this episode where 1 is very poor quality and 10 is excellent quality?

Rating out of 10: ______/10

1. What factors positively affected your audio description for this episode? Check all that apply.
   - Describer’s pace
   - Language & vocabulary used
   - Style of description
   - Fit of Description to Show
   - Amount of information conveyed.
   - Quality of description
   - Other factor(s), please specify: __________________
2. What factors negatively affected your rating of this episode? Check all that apply.

Describer

- - Tone of voice
  - Pace was too slow
  - Pace was too fast

Language &Vocabulary

- - Too simple
  - Too complex

Quantity

- - Too much description
  - Too little description

Style of Description

- - Did not match show
  - Deviated from conventions
  - Obtrusive
  - Interrupted enjoyment of show
  - Other factor(s), please specify: __________________

1. Please provide any additional comments on the show or the description.

**Death Comes to Town**

**Post-Questionnaire (after all episodes have been viewed)**

Thank you for your participation in this study. We appreciate your time and effort and your input in very valuable to the development of audio description in Canada. This questionnaire asks you to summarize your opinions and overall experience with the audio description and the show. It should only take about 10 minutes to complete.

1. Please indicate, in general, how entertained you were by the show series.
   - Very Entertained
   - Entertained
   - Uncertain
   - Not really entertained
   - Not entertained at all
2. Please indicate, in general, how entertained you were by the audio description of the show series.
   - Very Entertained
   - Entertained
   - Uncertain
   - Not really entertained
   - Not entertained at all
3. Please rate how similar the style of the language used by the describer matched that used in the program to that which was used in the show?
   - Very Similar
   - Somewhat similar
   - Uncertain
   - Somewhat dissimilar
   - Completely dissimilar
4. Please rate your level of agreement with the following statements (use agree, somewhat agree, didn’t care, somewhat disagree or disagree)
5. I looked forward to the getting each episode.
6. I was distracted by the description.
7. I disliked the series in general.
8. I talked to my friends about the show.
9. The description gave me enough information about the onscreen action/plot to make the show understandable for me.
10. I wanted more information about the characters.
11. The description gave me enough information about the setting.
12. I was bored with most episodes.
13. We have often though that people would be interested in listening to an audio only version of a television show while participating in activities that require their visual attention (driving, running, etc.) Please rate how likely you would be to purchase and listen to an audio only version of your favorite television show.
    - I would be interested in purchasing an audio only track of my favorite television show.
    - Not very likely to purchase an audio only track of my favorite television show.
    - I would not be interested in purchasing an audio only track of my favourite television show.
14. In general, what aspects of the description for Death Comes to Town did you like? Why?
15. In general, what aspects of the description for Death Comes to Town did you dislike? Why?
16. If you have any other comments that you would like to share, please include them here.
